# Influence of Maximum Nominal Size on Macro- and Meso-Mechanical Properties of Cement-Stabilized Macadam

**DOI:** 10.3390/ma19081611

**Published:** 2026-04-17

**Authors:** Wei Zhou, Changqing Deng, Huiqi Huang

**Affiliations:** 1 College of Civil and Architectural Engineering, Shaoyang University, Shaoyang 422000, China; zhouuwei@126.com; 2 Key Laboratory of Green Construction and Intelligent Monitoring in Southwestern Hunan for Regular Institutions of Higher Education of Hunan Province, Shaoyang 422000, China; 3 Hunan Engineering Research Center for Bamboo Fiber Construction Materials, Shaoyang 422000, China; 4 Key Laboratory for Special Area Highway Engineering of the Ministry of Education, Chang’an University, Xi’an 710064, China; 5 Shaanxi Transportation Holdings Group Co., Ltd., Xi’an 710061, China; 13323698951@163.com

**Keywords:** cement-stabilized macadam, discrete element method, force chain network, meso-mechanics, gradation design, uniaxial compression test

## Abstract

**Highlights:**

- A combined laboratory—DEM framework was established to clarify the macro-meso mechanical mechanisms of NMAS in cement-stabilized macadam.
- Increasing NMAS significantly enhances compressive strength and deformation capacity by forming a stronger and more efficient force-chain skeleton dominated by coarse aggregates.
- Larger NMAS delays micro-crack initiation, reduces crack propagation and total crack number, thereby improving damage resistance.

**Abstract:**

The nominal maximum aggregate size (NMAS) plays a critical role in determining the mechanical performance of cement-stabilized macadam (CSM), yet its meso-mechanical influence mechanism remains insufficiently understood. In this study, three skeleton-dense CSM mixtures with different NMAS values were designed, and a combined experimental–numerical approach was adopted to investigate the macro- and meso-scale mechanical behavior. Uniaxial compression tests and aggregate crushing value tests were conducted to evaluate strength development and load-transfer characteristics, while a three-dimensional discrete element method (DEM) model incorporating realistic aggregate morphology was established to analyze the evolution of contact forces and crack propagation. The results show that increasing NMAS significantly improves the mechanical performance of CSM. Compared with CSM-30, the 7-day compressive strength of CSM-40 and CSM-50 increased by approximately 10.3% and 37.3%, respectively. The stress–strain response indicates that mixtures with larger NMAS exhibit higher stiffness and a higher strain. At the meso-scale, a larger NMAS promotes the formation of a more efficient force-chain network dominated by coarse aggregates. Strong contacts were predominantly carried by aggregates larger than 9.5 mm, and in CSM-50, the proportion of strong contacts in the 37.5–53 mm fraction exceeded 90%, indicating that the largest particles likely form the primary load-bearing skeleton. In addition, increasing NMAS delayed crack initiation, reduced crack propagation rate, and decreased the total number of cracks at failure. These findings demonstrate that macroscopic strength improvement is closely associated with meso-scale optimization of the aggregate skeleton and enhanced load-transfer efficiency. This study provides a mechanistic basis for NMAS selection and gradation optimization in semi-rigid base materials.

## 1. Introduction

Cement-stabilized macadam (CSM) is widely used in high-grade highways and heavy-duty pavement structures due to its excellent mechanical performance and cost-effectiveness [1,2,3,4]. Nevertheless, its service life is still constrained by problems such as mechanical deterioration, reflective cracking, and fatigue failure under the combined effects of long-term cyclic loading and environmental exposure [5,6,7,8]. With the rapid expansion of transportation infrastructure and the continuous increase in traffic demand, higher performance requirements are being imposed on pavement materials. Consequently, a deeper understanding of the mechanical behavior of CSM and the factors governing its performance has become increasingly important.

Among the various influencing factors, aggregate gradation plays a crucial role in determining the mechanical properties of CSM [9,10,11]. Existing research has conducted extensive explorations in the areas of gradation types, design methods, evaluation indicators, and gradation development for different materials [12,13]. In terms of gradation types, studies have shown that skeleton-dense structures can effectively form stable skeletons, significantly enhancing the mechanical properties and crack resistance of materials. Tian found that a strong interlocking skeleton gradation composed of three types of coarse aggregates—19–31.5 mm, 9.5–19 mm, and 4.75–9.5 mm—in a mass ratio of 5:3:2 significantly improved resistance to shrinkage deformation [14]. Ji demonstrated that an interlocking gap gradation, achieved by controlling the passing rate of key sieve sizes, simultaneously enhances both mechanical properties and crack resistance [15]. Kong proved that an interlocking dense gradation designed using the Discrete Element Method enables recycled brick-concrete aggregates to form an effective force chain transmission system [16]. In terms of gradation design methods, Liu systematically analyzed the performance variation patterns of five gradation types from coarse to fine using the uniform interpolation method, discovering that the maximum dry density follows a quadratic curve relationship with gradation [17]. Kong, on the other hand, progressively designed an optimal interlocking dense gradation based on the Discrete Element Method [16]. Regarding evaluation systems, Li innovatively proposed mesoscopic evaluation indicators such as the skeleton denseness index and skeleton stability index, verifying their strong correlation with macroscopic mechanical strength [18]. Liang used CT scanning technology to reveal the distribution characteristics of internal voids in different gradations. In the development of gradations for different materials, research has covered a variety of special materials [13]. Zeng’s study on coral aggregates found that they only form a strong, suspended dense structure and cannot establish an effective skeleton, identifying an optimal gradation with a maximum particle size of 53 mm and a fine aggregate content of 45% [19]. Khamseh explored the cement stabilization of iron ore tailings, finding that a cement content of 5–10% significantly improves their mechanical properties [20]. Li also compared the performance differences in different permeable base gradations, such as dense and skeleton-void types [21]. Furthermore, studies have found that 4.75 mm is commonly used as the demarcation point between coarse and fine aggregates, and the vibration compaction method is more effective than the static pressure method in ensuring the realization of gradation design [22]. Although many studies have examined the influence of gradation type and mix-design methods on the performance of CSM, NMAS has rarely been isolated as a governing structural variable within skeleton-dense gradation systems. Moreover, research specifically addressing the impact of NMAS on mechanical performance remains scarce. In particular, there is a notable lack of quantitative force chain analysis and mechanistic interpretations that account for variations in NMAS, leaving the specific role of NMAS in controlling skeleton formation, coarse-fine aggregate load sharing, and the resulting mechanical response insufficiently clarified.

In recent years, with advancements in computational technology, the Discrete Element Method (DEM) has emerged as an effective tool for studying the meso-mechanical mechanisms of granular materials due to its ability to accurately simulate contact mechanics [23,24,25]. By constructing numerical models of particle assemblies, DEM can intuitively characterize the spatiotemporal evolution of the force chain network and quantitatively analyze load transfer paths [26,27]. Currently, DEM has demonstrated unique advantages in the study of materials such as asphalt mixtures and unbound granular layers [28,29,30]. Zhao employed discrete element theory to dynamically simulate uniaxial compression tests on cement-stabilized crushed stone, analyzing the dynamic evolution of microstructures and parameters [31]. Shi developed a CSM compaction model using DEM combined with SVM-DE algorithms, quantifying the impact of environmental factors on microscopic parameters [32]. Zhao established a DEM fatigue model that accounts for initial micro-defects, revealing the influence of defect size and distribution on fatigue crack propagation [33]. Ling utilized a BPNN-PSO machine learning approach to significantly enhance the accuracy and efficiency of CSM mesoscopic parameter inversion [34]. He investigated the anisotropy of coarse aggregates and their volume fraction’s regulatory effect on the mixture’s resistance to deformation through DEM, determining the optimal skeleton structure [35]. However, the application of the DEM to probe into the meso-scale mechanical behavior and its evolution in CSM under the influence of varying NMAS represents a significant research vacancy. Specifically, how NMAS quantitatively regulates the force-chain network distribution, the load-transfer mechanism between coarse and fine aggregates, and its impact on crack propagation are key questions that remain to be elucidated.

Based on this, the study adopts a combined approach of laboratory experiments and the DEM to investigate the influence of the NMAS on the macro- and meso-mechanical properties of CSM. By quantitatively analyzing contact forces, contact numbers, and crack evolution characteristics, the study provides an interpretation of how particle-size variation may regulate the macroscopic strength of CSM from a meso-mechanical perspective. The research can provide a scientific basis for the gradation design of high-performance CSM base courses, and the established discrete element analysis method can be extended to the performance study of other granular composite materials.

## 2. Materials and Methods

### 2.1. Materials and Gradations

The employed Portland cement P.O 42.5 was produced in Xi’an City, Shaanxi Province, China. Table 1 lists the technical indicators.

The limestone employed was obtained from Bao Ji City, Shaanxi Province, China. Table 2 lists the technical specifications.

**Figure 1:**
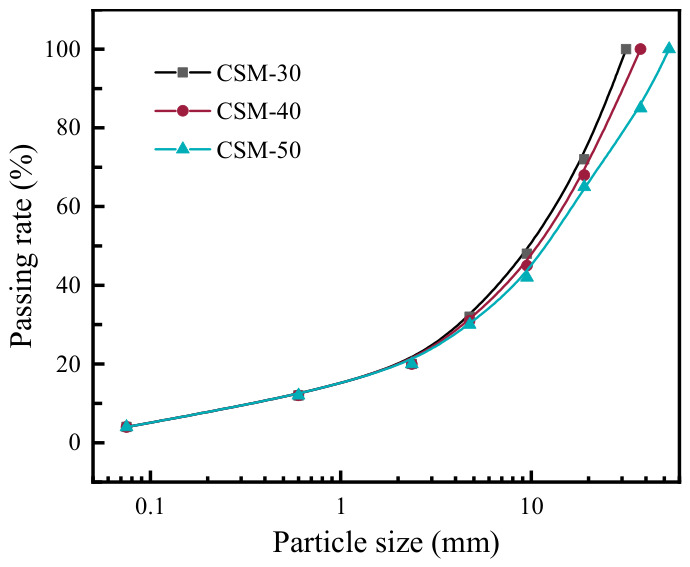
Gradation Curves.

Three skeleton-dense gradations with different NMAS were designed, with their gradation curves shown in Figure 1. The exact passing percentages corresponding to the three gradation curves are listed in Table 3.

### 2.2. Uniaxial Compression Test

**Figure 2:**
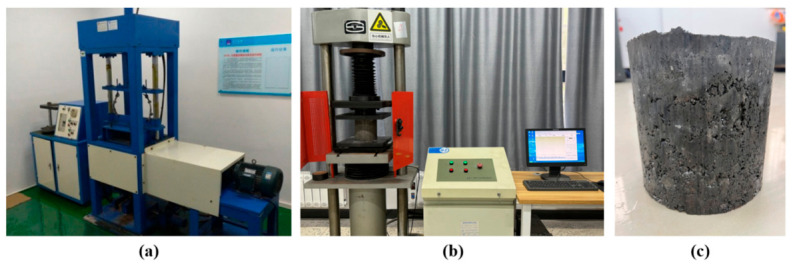
Test equipment and specimens: (**a**) Vibration equipment; (**b**) Pressure testing machine; (**c**) Specimen.

The specimens were prepared using the vibration compaction method, with dimensions of 150 mm in height and 150 mm in diameter. The cement content was 4% by mass of dry aggregate. After compaction, the specimens were sealed for 24 h, demolded, and then cured in a standard curing room at 20 ± 2 °C and a relative humidity of 95% until the designated ages of 7, 14, 28, 60, and 90 days. Before the unconfined compression test, the specimens were soaked in water at 20 ± 2 °C for 24 h. For each mixture and curing age, 6 replicate specimens were tested, and both the mean and standard deviation were reported to reflect variability between replicates. The unconfined compressive strength test was conducted at a loading rate of 1 mm/min. Test equipment and specimens are shown in Figure 2. The compressive strength without the lateral limit is calculated according to Equation (1):

(1) $$P=\frac{R_{c}}{A}$$

where *R_c_* is the unconfined lateral compressive strength (MPa), *P* is the maximum pressure of the specimen at the time of damage (N), and *A* is the specimen cross-sectional area (mm^2^).

### 2.3. Uniaxial Compression Numerical Test

#### 2.3.1. Numerical Model Construction

**Figure 3:**
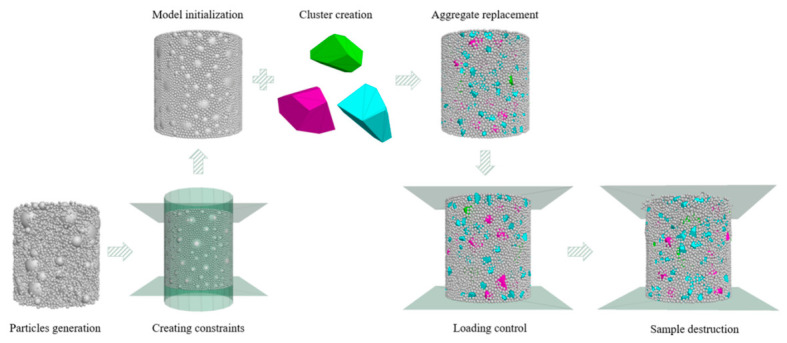
Numerical test construction flowchart.

This study established three-dimensional cylindrical uniaxial compression numerical models of CSM with different NMAS using PFC^3D^ (version 6.0) software, as shown in Figure 3. The detailed modeling procedure was as follows: (1) Discrete element generation: CSM specimens were generated within a cylindrical domain using the ball distribution method according to particle quantities calculated from actual gradations. To simulate the mechanical behavior of real aggregates and cement mortar, appropriate density and damping coefficients were assigned to different particles, with those smaller than 2.36 mm defined as the cement mortar matrix. This simplified method is widely adopted because the very fine fraction in cement-stabilized aggregates primarily functions as a pore-filling component embedded within the cement paste, rather than participating in the main stone-stone skeleton like the coarse aggregates. Additionally, explicitly accounting for all fine particles and hydration products would lead to excessive computational cost [25,29]. (2) Model initialization: Upper and lower loading plates and a cylindrical lateral confinement boundary were created using the wall generate command, after which particles extending beyond the boundaries were deleted. The system was then equilibrated for 2000 calculation steps to reach initial stress equilibrium and a stable state. (3) Clump simulation of coarse aggregates: STL geometry files were imported to replace coarse aggregate particles with clumps, accurately simulating the morphology of irregular aggregates. The volume error during this process was strictly controlled within ±1% to ensure geometrical accuracy. (4) Loading and failure simulation: Uniaxial compression loading was applied by controlling wall displacement until macroscopic failure occurred, thereby simulating the complete process of a physical uniaxial compression test.

**Figure 4:**
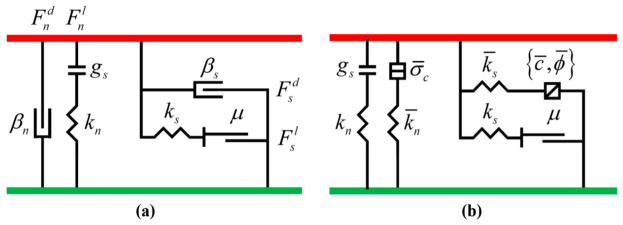
Schematic of the Contact Model: (**a**) The rolling resistance linear contact model; (**b**) The parallel bond model.

In the numerical model, the macroscopic properties of the mixture were characterized by defining the contact relationships between particles. To improve computational efficiency, the contact types in the model were simplified into three categories: contacts between coarse aggregate particles and walls (ball-facet), contacts between coarse aggregate particles (ball-ball), and contacts between mortar particles (mortar). The uniaxial compression behavior of CSM was simulated by assigning distinct mechanical models to these three contact types. The rolling resistance linear contact model and the parallel bond model, both in PFC^3D^ software, were employed to simulate the aforementioned contact relationships. The rolling resistance linear model was assigned to aggregate-dominated contacts, including aggregate–aggregate and aggregate–wall interactions, because it can effectively represent frictional sliding, particle interlocking, and rotational resistance among irregular coarse aggregates. These mechanisms are essential for describing the skeletal load-transfer behavior of CSM. The parallel bond model was assigned to mortar-dominated contacts because it can simulate the finite tensile and shear resistance of the cemented mortar phase before bond failure. Therefore, the combined use of these two contact models is considered suitable for reproducing the coupled frictional–cemented mechanical behavior of real CSM mixtures [37]. Schematic diagrams of these two models are presented in Figure 4.

**Figure 5:**
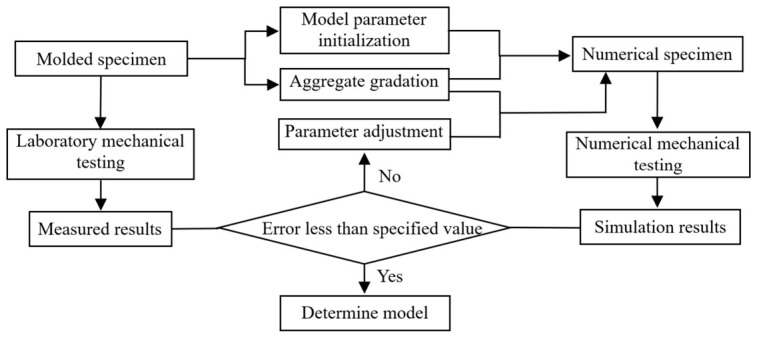
Numerical test model parameter calibration process.

The model parameters were calibrated through an iterative procedure combining literature-based initial estimates and trial-and-error adjustments [32], as illustrated in Figure 5. Specifically, a reasonable parameter range was first determined based on previous DEM studies of cement-stabilized granular materials. The parameters were then gradually adjusted until the simulated stress–strain curves matched the experimental responses with satisfactory accuracy. The final set of parameters reported in Table 4 corresponds to the optimal overall agreement between the numerical and experimental responses.

#### 2.3.2. Model Reliability Verification

**Figure 6:**
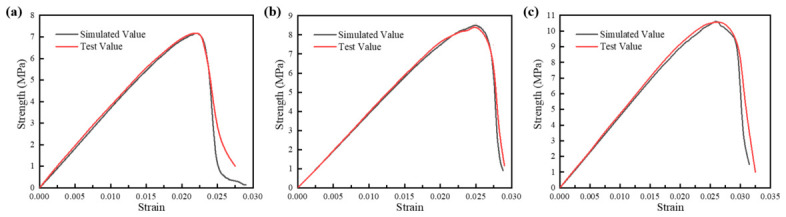
Stress–strain curve: (**a**) CSM-30; (**b**) CSM-40; (**c**) CSM-50.

To validate the reliability of the established discrete element model, a comparative analysis was conducted between the numerical simulation stress–strain curves and laboratory test results for three groups of CSM specimens with different gradations, as shown in Figure 6. The results demonstrate that the numerical simulation curves closely match the experimental curves throughout the loading process up to the peak stress point, with both the peak strength and its corresponding strain showing strong agreement with experimental values. The RMSE values for the three gradations shown in Figure 6a–c were estimated to be approximately 0.40 MPa, 0.15 MPa, and 0.28 MPa, respectively.

Although some discrepancies are observed in the post-peak stage, this limitation does not invalidate the present meso-mechanical analysis. The main purpose of the DEM model is to capture the evolution of force chains, crack initiation, and comparative damage development up to peak stress and in the early post-peak stage, where the numerical and experimental responses remain in close agreement. The post-peak mismatch is mainly attributed to the simplified representation of mortar heterogeneity, localized softening, and crack coalescence in the DEM model. Therefore, the model is considered reliable for comparative analysis of crack evolution mechanisms among different NMAS mixtures, while caution should be exercised when interpreting the exact post-peak softening response.

## 3. Results

### 3.1. Macroscopic Mechanical Properties Analysis

#### 3.1.1. Mechanical Strength Analysis

**Figure 7:**
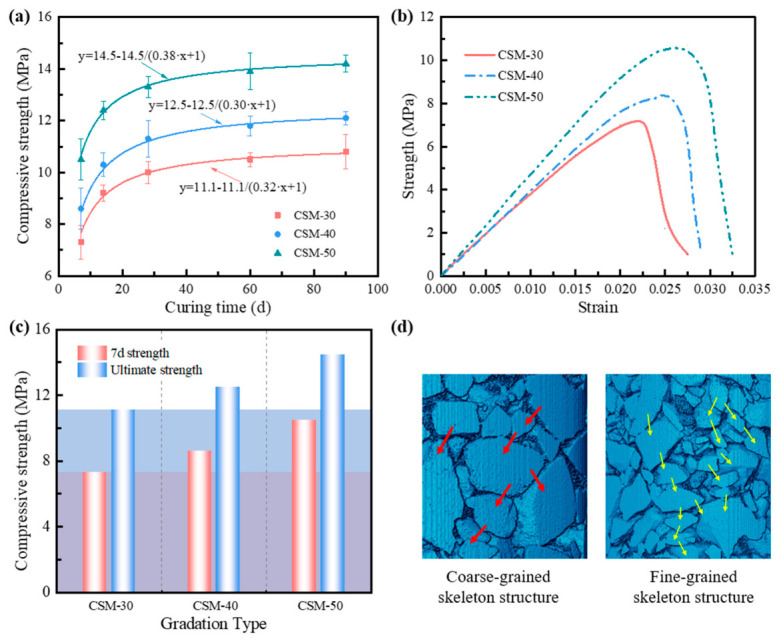
Characterization of mechanical properties for CSM: (**a**) Development of compressive strength; (**b**) Stress–strain curves; (**c**) Comparison of mechanical strength; (**d**) Schematic diagram of the mechanical enhancement mechanism.

The compressive strength evolution of the three CSM gradations at different curing ages is presented in Figure 7a. For each mixture and curing age, six replicate specimens were tested, and the results are reported as mean ± standard deviation. As observed, the strength of all mixtures exhibits a rapid initial increase followed by a gradual stabilization with prolonged curing time. Moreover, the standard deviations remain relatively small, indicating good repeatability of the test results. All measured mechanical strength values exhibited standard deviations not exceeding 0.8 MPa. Statistical significance analysis was further conducted to compare the strength differences among mixtures with different NMAS values, and the results confirmed that the strength increase with NMAS was statistically significant (*p* < 0.05). Notably, the CSM with a larger NMAS consistently demonstrated superior strength performance throughout the entire curing period. This trend is further corroborated by the stress–strain curves obtained at 7 days (Figure 7b). The mixture with the larger NMAS displayed a higher strain at peak stress, which should not be interpreted as indicating greater deformation capacity. Instead, the stronger and more stable coarse-aggregate skeleton delayed the onset of unstable cracking, allowing the specimen to sustain greater pre-peak deformation before reaching the maximum load. However, once the main crack formed, the post-peak response still exhibited a relatively abrupt stress drop, indicating that the material remained brittle after peak loading. To quantitatively characterize the age-dependent strength evolution, an empirical asymptotic growth function, y = a − a/(b*x + 1), was used to fit the experimental data, where x is the curing time, and y is the compressive strength [1,38]. This function was selected because it captures the typical strength-development pattern of cement-stabilized materials, namely a rapid increase at early curing ages followed by a gradual approach to a stable value at later ages. Therefore, the model is employed here as a practical fitting function for trend characterization rather than as a fundamental mechanistic law.

The parameter a in this equation represents the theoretical asymptotic peak strength that the CSM can achieve over an infinite curing time, thus serving as a crucial indicator for evaluating the ultimate mechanical capacity of the material. A direct comparison of the 7-day compressive strength and the fitted ultimate strength (a) for the three gradations is shown in Figure 7c. The results indicate that the larger NMAS mixture (CSM-50) tends to exhibit higher short-term and long-term strength metrics.

To elucidate the mechanical mechanism behind the performance improvement of the larger NMAS mixture, a comparative schematic diagram of stress transmission in coarse and fine-grained skeleton structures was constructed (as shown in Figure 7d). The superior performance of the larger-NMAS mixture can be more directly attributed to the formation of a stronger and more continuous aggregate skeleton, which enables more efficient load transfer through coarse aggregate contacts. The skeleton formed by coarse-grained establishes a more direct force chain network, as indicated by the thick red arrows in Figure 7d; the stress is transmitted through stronger and more efficient paths. In contrast, although finer NMAS mixtures exhibit a dense-graded characteristic, their force chains tend to be more numerous, relatively weaker, and more tortuous, which could lead to less efficient stress distribution under high loads. In addition, considering that a lower aggregate specific surface area at the same cement content may favor the development of a thicker paste coating, the aggregate–mortar interfacial bonding may also be improved [36]. This enhances the bond strength at the aggregate-mortar interface and effectively suppresses the initiation and propagation of micro-cracks, thereby synergistically improving the macroscopic mechanical strength and deformation resistance of the material.

#### 3.1.2. Load Contribution Analysis

**Figure 8:**
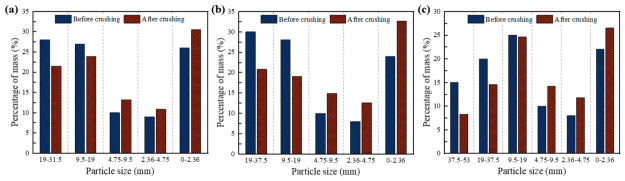
Results of aggregate particle size changes before and after crushing tests: (**a**) CSM-30; (**b**) CSM-40; (**c**) CSM-50.

The crushing value test was employed to evaluate the resistance of coarse aggregates in CSM to fragmentation, reflecting the particle stability of the material under load. This test simulates the transmission of external load through an aggregate skeleton, with the extent of particle breakage serving as an indirect indicator of load-transfer efficiency among aggregates. The changes in particle size distribution before and after crushing were analyzed through sieving, with the results presented in Figure 8. As shown in the figure, the mass proportion of coarse aggregates larger than 19 mm decreased in all three mixtures after the crushing test, indicating significant breakage of particles in this size range. This observation revealed that these larger particles play a predominant load-bearing role, making them more susceptible to crushing under load. Furthermore, the mass proportion of the 9.5–19 mm aggregates in CSM-50 remained nearly unchanged. This can be attributed to two factors: first, some particles in this range originated from the breakage of larger aggregates during the test; second, this size fraction bears relatively lower contact forces within the skeleton, allowing it to remain stable under crushing load. In contrast, the significant reduction in the 9.5–19 mm fraction in both CSM-40 and CSM-30 suggests that this fraction plays a more critical load-bearing role in these finer gradations. Although the crushing value test represents a simplified laboratory loading condition rather than the full complexity of field traffic action, it still provides a comparative indicator of the aggregate skeleton’s resistance to particle breakage under concentrated compressive loading. From an engineering perspective, a mixture in which the key load-bearing aggregate fractions remain relatively stable during crushing may help maintain a more robust skeleton structure in service, potentially contributing to improved resistance against traffic-induced deformation and structural deterioration.

### 3.2. Meso-Scale Mechanical Characterization

#### 3.2.1. Contact Force Characteristic Analysis

**Figure 9:**
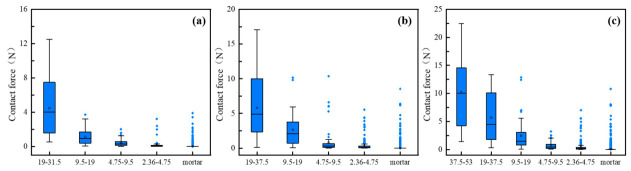
Contact force distribution of aggregate particles with different sizes: (**a**) CSM-30; (**b**) CSM-40; (**c**) CSM-50.

Figure 9 illustrates the contact force distribution across aggregate particle sizes for different gradations. As the particle size increases, the distribution range of contact forces progressively widens, with the largest aggregates exhibiting the broadest distribution. This indicates that the coarse aggregate particles form the primary load-bearing skeleton, transmitting the majority of the external load. Notably, with the increase in NMAS, the 37.5–53 mm aggregates in CSM-50 show the widest distribution range, which may indicate the dominant skeletal role of the largest-sized coarse aggregates. In contrast, the contact forces between fine aggregates and the mortar exhibit a narrower distribution range but with a substantial number of outliers, suggesting the presence of localized stress concentrations within the mortar matrix.

**Figure 10:**
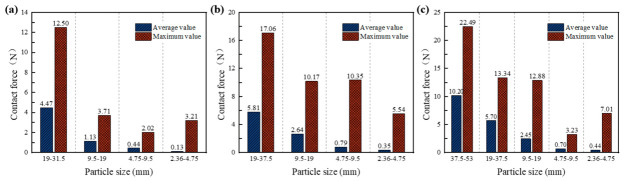
Statistical results of maximum and average contact forces between aggregate particles: (**a**) CSM-30; (**b**) CSM-40; (**c**) CSM-50.

To further quantify the role of aggregates in load transmission, Figure 10 analyzes the maximum and average contact forces of aggregate particles larger than 2.36 mm. Although the mortar matrix also participates in internal force transmission, its role in the primary load-bearing skeleton is considered secondary to that of coarse aggregate contacts in the present DEM framework. Therefore, the following analysis focuses on aggregate-related contact forces to better characterize the dominant skeletal load-transfer mechanism. Nevertheless, the quantitative contribution of the mortar phase warrants further sensitivity analysis in future studies. The results indicate that larger aggregates exhibit both higher average and maximum contact forces. This trend unequivocally demonstrates the existence of a hierarchical load-transfer structure within the material, wherein larger aggregates form the skeletal framework of the force chain network and dominate the transmission of external loads. Larger particles generally contribute more to resisting the applied load, though local interactions and specimen heterogeneity may modulate this effect.

Although the present DEM model does not explicitly discretize the interfacial transition zone (ITZ) as an independent phase, its mechanical influence should not be neglected. In cement-stabilized materials, the ITZ is generally the weakest local region and may affect local stress redistribution and crack initiation. In the current model, this effect is implicitly reflected by the calibrated bond stiffness and bond strength assigned to the mortar-dominated contacts. Therefore, the present interpretation focuses on aggregate–aggregate force transmission as the primary skeleton mechanism, while the contribution of the ITZ is discussed as a secondary but important factor that warrants direct characterization in future work.

#### 3.2.2. Contact Number Characteristics Analysis

**Figure 11:**
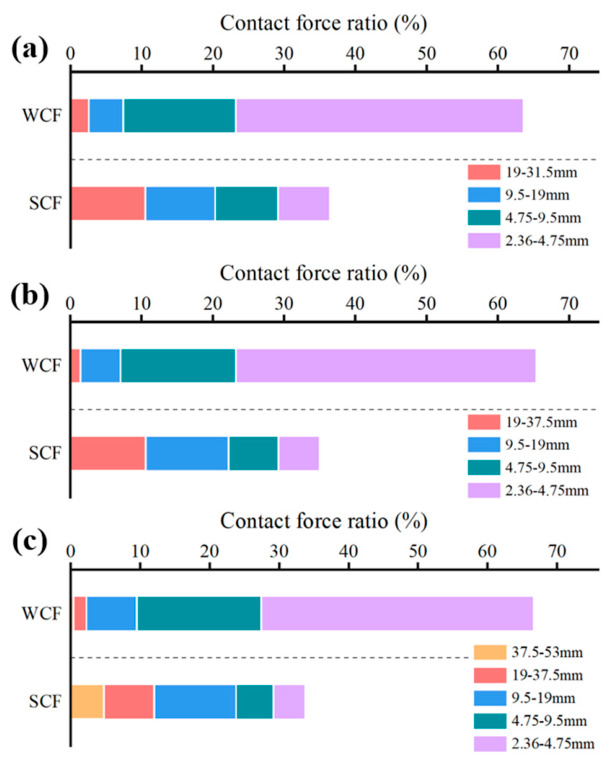
Number of contact forces for different particle sizes in each gradation: (**a**) CSM-30; (**b**) CSM-40; (**c**) CSM-50.

Figure 11 shows the distribution of strong and weak contact forces across aggregate particles of different sizes. Here, strong and weak contacts are defined relative to the average contact force; values above the average are classified as strong contact force (SCF), while those below are considered weak contact force (WCF). The analysis indicates that, across all gradations, strong contacts are primarily borne by aggregates larger than 9.5 mm, reflecting to some extent the important role of aggregates above 9.5 mm in load transfer. In contrast, the finer aggregates in the ranges of 2.36–4.75 mm and 4.75–9.5 mm are more likely to provide weak contacts, with their structural role tending toward void filling and skeleton stabilization rather than dominating load transfer. This mechanically explains why excessive content of these fractions should be avoided in mix design, as it may interfere with the formation of an effective primary skeleton. Furthermore, in gradations with a smaller NMAZ, the proportion of strong contacts contributed by aggregates below 9.5 mm increases relatively. This suggests that when the coarse skeleton is insufficient, finer aggregates may transition from a filling role to partially undertake secondary load-bearing functions.

**Figure 12:**
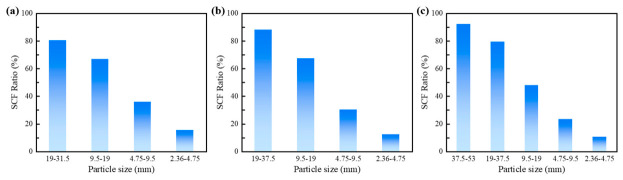
Proportion of strong contact forces: (**a**) CSM-30; (**b**) CSM-40; (**c**) CSM-50.

Figure 12 presents a statistical analysis of the proportion of strong contacts for each aggregate fraction. The results indicate that the load transfer paths within the material are predominantly governed by coarse aggregates, and this dominance intensifies with increasing particle size. In the CSM-50 gradation, the proportion of strong contacts for the 37.5–53 mm aggregates exceeds 90%, providing meso-scale evidence that these largest particles form the primary load-bearing skeleton and serve as the critical pathway for sustaining the vast majority of the applied load.

#### 3.2.3. Crack Evolution Characteristics Analysis

**Figure 13:**
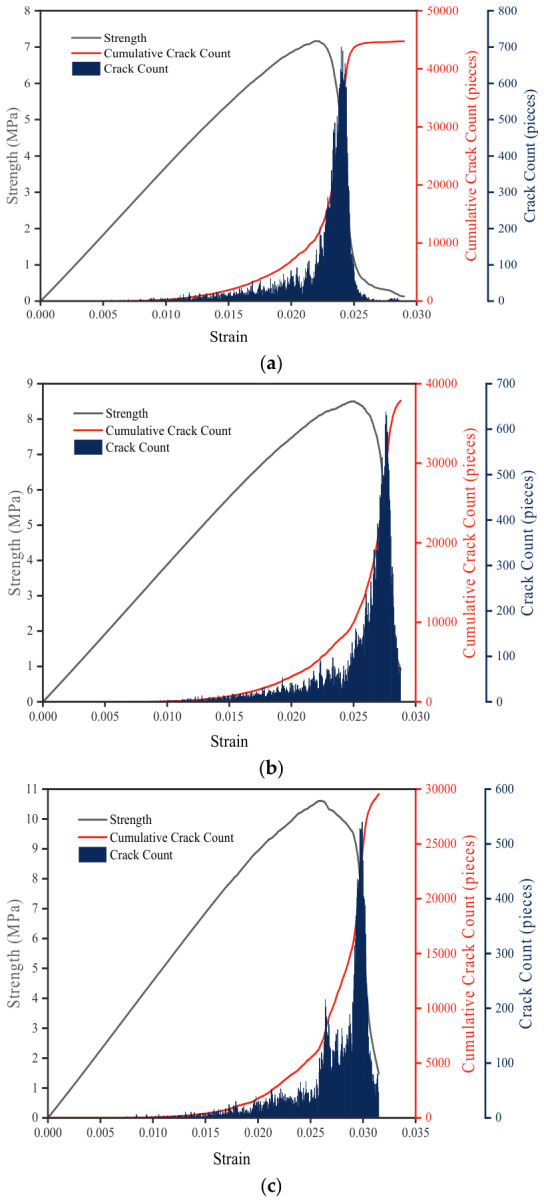
The development of micro-crack numbers: (**a**) CSM-30; (**b**) CSM-40; (**c**) CSM-50.

Figure 13 illustrates the development of micro-crack numbers with strain for the three CSM mixtures. It can be clearly observed that compared to specimens with smaller NMAS, those with larger NMAS exhibit significantly delayed micro-crack initiation, i.e., a higher strain threshold for crack formation. During the subsequent stable crack growth stage, the crack growth curve of the larger NMAS specimen shows a gentler slope, indicating a slower crack propagation rate. More importantly, the total number of cracks at macroscopic failure is significantly lower in these specimens. These findings demonstrate that increasing NMAS improves crack resistance and suppresses damage accumulation under monotonic uniaxial compression. However, since fatigue, cyclic loading, and environmental actions were not considered in the present study, these findings should not be directly interpreted as proof of long-term durability enhancement without further experimental verification.

**Figure 14:**
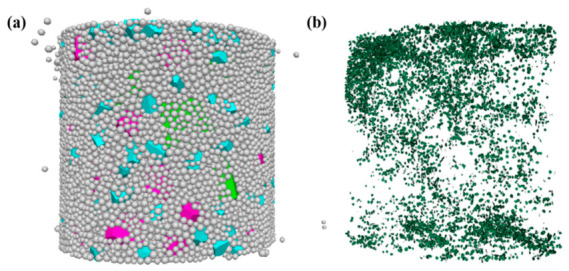
Crack morphology from the uniaxial compression numerical test: (**a**) Schematic diagram of virtual specimen failure; (**b**) Schematic diagram of crack distribution.

**Figure 15:**
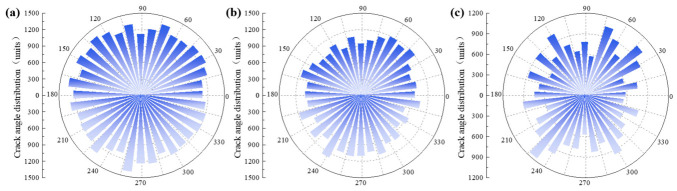
Characteristics of crack angular distribution: (**a**) CSM-30; (**b**) CSM-40; (**c**) CSM-50.

To further investigate the influence of the NMAS on crack distribution characteristics in CSM, the crack patterns at the peak stress of uniaxial compression numerical tests were extracted, as shown in Figure 14. Rose diagrams of crack statistics for the three mixtures are further presented in Figure 15. The results reveal notable differences in crack distribution with increasing NMAS. In the CSM-30 specimen, microcracks initiate randomly under shear, resulting in dispersed crack orientations and a near-circular rose diagram. As the NMAS increases, crack orientations become more concentrated, forming a distinct bimodal petal-shaped pattern in the rose diagram. In CSM-50, the skeleton formed by coarse aggregates appears to influence the internal stress field, potentially guiding the initiation of microcracks along the principal stress direction of 30–80°. During propagation, these cracks are obstructed by surrounding large aggregates, forcing them to deflect, bypass, or terminate. Consequently, their propagation paths and directions are strongly confined within the skeletal pores. This mechanism not only delays crack coalescence but ultimately results in the formation of a single, localized macroscopic crack. In contrast, the fine aggregates in CSM-30 disperse stresses through numerous contact points, allowing microcracks to initiate randomly and propagate freely in multiple directions. This eventually leads to a diffuse network of microcracks, exhibiting a more ductile failure mode.

## 4. Conclusions

This study investigates the influence of the NMAS on the macro- and meso-mechanical properties of CSM through an integrated approach of laboratory experiments and DEM simulations. The primary findings are summarized as follows:

(1) At the macro-scale, a clear trend of enhanced mechanical performance with increased NMAS was established. Specimens with a larger NMAS exhibited significantly higher unconfined compressive strength, greater stiffness, and a higher deformation capacity at failure. The crushing value tests further corroborated that the coarse aggregate skeleton in larger NMAS mixtures possessed superior stability and load-bearing capacity under compressive loading.
(2) DEM analysis revealed that a larger NMAS promoted the formation of a more robust and heterogeneous force-chain network. Coarse aggregates, particularly those exceeding 9.5 mm, acted as the dominant skeletal framework, bearing disproportionately higher average and maximum contact forces. The statistical distribution of strong contacts unequivocally identified these particles as the primary load-transfer paths.
(3) The evolution of micro-cracks indicated that mixtures with a larger NMAS tended to exhibit delayed crack initiation strain, a slower crack propagation rate, and a lower total number of cracks at failure, although variability exists between specimens. This observed improvement in damage tolerance may be related to stress distribution within the coarse skeleton and the potential barrier effect of large aggregates, which could influence crack propagation.
(4) This research provides a multi-scale perspective suggesting that increasing the NMAS may enhance macroscopic strength and deformation resistance of CSM through potential meso-scale structural optimization, subject to material and experimental limitations. Based on both macro- and meso-scale indicators, including compressive strength, stiffness, force-chain network efficiency, micro-crack development, and aggregate stability, CSM-50 was identified as the optimal gradation, providing a balanced combination of mechanical performance and practical applicability. These findings offer meso-mechanical evidence for gradation optimization, supporting the design of strong skeletal structures in cement-stabilized layers.
(5) Compared with existing methods for CSM analysis, the combined laboratory–DEM approach provides quantitative insights into force chain evolution and crack propagation, enabling more informed gradation design. Although the experimental and numerical results show generally consistent trends, limitations of this study include the simplified DEM representation of the mortar phase, implicit modeling of the ITZ, use of a single aggregate type, and quasi-static loading conditions. Future work should explore a wider range of materials, mixture designs, and environmental or cyclic loading.

## Figures and Tables

**Table 1:** Technical specifications of cement [36].

Test Index	Adequacy (%)	Coagulation Time (min)		Flexural Strength (MPa)		Compressive Strength (MPa)	
		Initial Coagulation Time	Final Coagulation Time	3 d	28 d	3 d	28 d
Test results	Qualified	428	526	4.2	7.2	16.9	40.3

**Table 2:** Technical specifications of aggregates.

Test Project	Measured Values of the Mentioned Technical Specifications of Limestone with Different Grain Sizes (mm)		
	20–30	10–20	5–10
Apparent relative density (g/cm^3^)	2.76	2.73	2.70
Needle flake particle content (%)	14.6	15.3	10.2
Water absorption rate (%)	0.43	0.88	1.01
Crushing value (%)	13.2		

**Table 3:** Gradation design of the three CSM mixtures.

Gradation Type	The Mass Percentage (%) of the Following Sieve Size (mm)								
	53	37.5	31.5	19	9.5	4.75	2.36	0.6	0.075
CTB-50	100	85	-	65	42	30	20	12	4
CTB-40	100	100	-	68	45	31	20	12	4
CTB-30	100	100	100	72	48	32	20	12	4

**Table 4:** Calibration results of microscopic contact parameters.

Model Type	Parameters	Value
Rolling resistance linear contact model	Modulus of elasticity (MPa)	500
	Rigidity ratio	1.5
	Coefficient of friction	0.5
Parallel bond model	Modulus of elasticity (MPa)	150
	Rigidity ratio	2.0
	Tensile strength (MPa)	2.9
	Cohesion (MPa)	3.5
	Internal friction angle (°)	30
	Coefficient of friction	0.5

## Data Availability

The original contributions presented in this study are included in the article. Further inquiries can be directed to the corresponding author.
